# A Turner syndrome neurocognitive phenotype maps to Xp22.3

**DOI:** 10.1186/1744-9081-3-24

**Published:** 2007-05-21

**Authors:** Andrew R Zinn, David Roeltgen, Gerry Stefanatos, Purita Ramos, Frederick F Elder, Harvey Kushner, Karen Kowal, Judith L Ross

**Affiliations:** 1 Eugene McDermott Center for Human Growth and Development and Department of Internal Medicine, The University of Texas Southwestern Medical School, Dallas TX, USA; 2 Cooper University Hospital, Robert Wood Johnson Medical School, Camden, NJ, USA; 3 MossRehab Research Institute, Albert Einstein Medical Center, Thomas Jefferson University, Philadelphia, PA,; 4 Department of Pathology, The University of Texas Southwestern Medical School, Dallas TX 75390, USA; 5 Biomedical Computer Research Institute, Philadelphia, PA, USA; 6 Department of Pediatrics, Thomas Jefferson University, Philadelphia, PA, USA

## Abstract

**Background:**

Turner syndrome (TS) is associated with a neurocognitive phenotype that includes selective nonverbal deficits, e.g., impaired visual-spatial abilities. We previously reported evidence that this phenotype results from haploinsufficiency of one or more genes on distal Xp. This inference was based on genotype/phenotype comparisons of individual girls and women with partial Xp deletions, with the neurocognitive phenotype considered a dichotomous trait. We sought to confirm our findings in a large cohort (n = 47) of adult women with partial deletions of Xp or Xq, enriched for subjects with distal Xp deletions.

**Methods:**

Subjects were recruited from North American genetics and endocrinology clinics. Phenotype assessment included measures of stature, ovarian function, and detailed neurocognitive testing. The neurocognitive phenotype was measured as a quantitative trait, the Turner Syndrome Cognitive Summary (TSCS) score, derived from discriminant function analysis. Genetic analysis included karyotyping, X inactivation studies, fluorescent in situ hybridization, microsatellite marker genotyping, and array comparative genomic hybridization.

**Results:**

We report statistical evidence that deletion of Xp22.3, an interval containing 31 annotated genes, is sufficient to cause the neurocognitive phenotype described by the TSCS score. Two other cardinal TS features, ovarian failure and short stature, as well as X chromosome inactivation pattern and subject's age, were unrelated to the TSCS score.

**Conclusion:**

Detailed mapping suggests that haploinsufficiency of one or more genes in Xp22.3, the distal 8.3 megabases (Mb) of the X chromosome, is responsible for a TS neurocognitive phenotype. This interval includes the 2.6 Mb Xp-Yp pseudoautosomal region (PAR1). Haploinsufficiency of the short stature gene *SHOX *in PAR1 probably does not cause this TS neurocognitive phenotype. Two genes proximal to PAR1 within the 8.3 Mb critical region, *STS *and *NLGN4X*, are attractive candidates for this neurocognitive phenotype.

## Background

Turner syndrome (45, X, TS, monosomy X) is the genetic disorder resulting from the absence of all or part of one X chromosome in females. The complex TS phenotype includes short stature, ovarian failure, and a characteristic neurocognitive profile [1]. Although severe developmental disorders do not predominate in TS, the risk of selective deficits in certain cognitive domains is substantially increased. Girls and women with 45, X TS commonly demonstrate deficits in visual-spatial abilities, visual-perceptual abilities, motor function, nonverbal memory, executive function and attentional abilities when compared to normal females matched for age, height, IQ, and socioeconomic status [2-9].

Both hormonal and genetic factors may influence the cognitive development of TS females. Hormonal influences are attributable to deficient ovarian production of estrogen, androgen, or both. Subtle estrogen effects on motor function and processing speed and androgen effects on working memory have been demonstrated [10-12]. However, many of the cognitive deficits characteristic of TS are consistent across a wide age range, including children as well as estrogen-replaced adults [4,11,12]. Furthermore, these deficits are generally not seen in females with premature ovarian failure and intact X chromosomes [13,14], suggesting a predominant role of genetic factors in the etiology of TS cognitive deficits.

The genetic abnormality in TS is determined by the absence of one copy of genes on the X chromosome. Most aspects of the TS phenotype, including the cognitive phenotype, are thought to be due to half-normal gene dosage, or haploinsufficiency, of X-linked genes that escape inactivation [15,16]. Although one X chromosome undergoes inactivation in normal females during early embryogenesis, about 15% of all X chromosome genes, mostly situated on the short arm (Xp), remain active to some degree on both X chromosomes [17]. Some of these genes have functional Y-linked homologs that are thought to balance their dosage between males and females. The pseudoautosomal region (PAR1), a 2.6 Mb interval at the tips of the X and Y short arms, is a plausible location for TS genes because PAR1 genes are all expressed at diploid dosage in both males and females [18,19]. One PAR1 gene, *SHOX *[Mendelian Inheritance in Man (MIM) 12865], has been implicated in TS short stature [20]. Whether any PAR1 genes contribute to other aspects of the TS phenotype is unknown.

One way to deduce the underlying genotype-phenotype relationships in TS is to compare the phenotypes of individuals missing various portions of one X chromosome in order to assign specific features to "critical regions." A trait maps to a region if deletion of that region accounts for the variance in that trait. This approach was used to map short stature to the *SHOX *gene on Xp [20] and ovarian failure to regions of the long arm (Xq) [21]. We have previously applied this phenotype mapping methodology to define genetic correlates of the cognitive phenotype in children with TS [22].

We used discriminant function analysis to derive a mathematically defined TS cognitive phenotype to test for association with deletions of specific regions of the X chromosome [22,23]. The initial results from 34 subjects with deletions of varying portions of Xp, mapped mostly by fluorescence in situ hybridization (FISH), identified a probable association between deletion of the distal ~10 Mb of Xp and the TS neurocognitive phenotype [22]. However, those conclusions relied upon identification of the TS neurocognitive phenotype in a small number of individual subjects; the sample was too small to permit rigorous statistical inference. The TS cognitive phenotype was defined as a dichotomous measure, rather than a quantitative trait, using a cutoff score. In addition, the population was heterogeneous, including both children and adults, and did not include any subjects with Xq deletions, who commonly receive the TS diagnosis.

In the present study, we attempted to confirm and expand the initial findings using 47 adult females with nonmosaic deletions distributed along both Xp and Xq. Potential sources of variation in cognitive outcome have been minimized by including only adults with similar estrogen-replacement status. In this study we treated the TS cognitive phenotype as a quantitative rather than a qualitative trait and compared the results of women with deletions of varying portions of the X chromosome to that of 45, X TS and normal controls. We hypothesized that an association exists between the defined TS neurocognitive phenotype and deletion of Xp22.3, which includes the distal 8.3 Mb of Xp.

## Methods

### Subjects

This study was approved by the Thomas Jefferson University Human Studies Committee and the UT Southwestern Institutional Review Board. Informed consent was obtained from all participants. Subjects, ages 17–55 years, were recruited from North American genetics and endocrinology clinics. We excluded subjects with sex chromosome mosaicism, ring X chromosomes, clinical features of autosomal aneuploidy in the case of unbalanced X;autosome translocations, or clinical diagnoses of Goltz [MIM 305600], Aicardi [MIM 304050], or MLS [MIM 309801] syndromes in the case of Xp22.3 deletions. Subjects with Verbal IQ (VIQ) < 69 were also excluded since significantly reduced VIQ is atypical for this population and, in the context of depressed spatial skills, implies generalized limitation of intelligence. Subjects with serum gonadotropin levels in the castrate range (≥ twice the upper limit of normal) and amenorrhea were determined to have ovarian failure. Some of the deletion subjects had normal menstrual histories and were evaluated in the follicular phase of their cycle. Subjects with ovarian failure were receiving standard estrogen replacement therapy (i.e. cycling with estrogen and progesterone).

### Cytogenetic and molecular analyses

Standard Giemsa-banded peripheral blood karyotypes were obtained for subjects not evaluated cytogenetically within the previous two years, with particular attention paid to the X chromosome. Lymphoblastoid cell lines were derived from blood samples by standard methods [24]. X inactivation pattern was measured from blood DNA using the androgen receptor methylation assay [25]. Metaphase spreads from blood or lymphoblastoid cells were used for FISH as previously described [22,26].

A variety of techniques were used to map deletions, including FISH [22,26], polymorphic microsatellite markers [27], and array comparative genomic hybridization (CGH) [28]. Genotyping data were interpreted as described previously [29]. Briefly, heterozygous markers were scored as not deleted, and markers showing loss of heterozygosity or non-inheritance of a parental allele were scored as deleted. Selected subjects whose deletions were initially mapped by FISH were restudied with microsatellite markers when parental DNAs became available. Breakpoints were inferred from data on relatives for subject 105 (blood sample unavailable) whose deletion was familial. Oligonucleotide array CGH was performed on male offspring of 430 and 702 known to carry the deletion by Nimblegen Systems, Inc. (Madison, WI) using an X chromosome tiling array (Catalog B3754001-00-01, one probe every 340 bp on average) and pooled normal male reference DNA (Promega Corp., Madison, WI). Probe signal intensities were averaged over a 4 kilobase window (~12 adjacent probes) and copy number changes detected using a circular binary segmentation algorithm [30]. Map locations of markers are based on the UC Santa Cruz Genome Browser, March 2006 Human Genome Assembly.

### Derivation of the TSCS score

All cognitive evaluations were administered and scored by psychometricians who were unaware of specific karyotype results. We first performed discriminant function analysis using the results of the battery of cognitive tests (Table 1) on populations of nonmosaic, 45, X TS subjects (n = 94) and age-, VIQ-, and socioeconomic status (SES)-matched normal female controls (n = 103), ages 17.0–55.0 years. The populations were randomly divided in halves to form generative and prospective replication samples. The analysis used the Mahalanobis distance formula to maximize the n-dimensional distance between group centroids to weigh the variables in order to optimally separate the TS and control generative samples. The resulting formula yielded the TS cognitive summary (TSCS) score. The components of the TSCS score and the weighting coefficients are indicated in Table 1. The mean TSCS scores differed significantly for the TS and control populations (53 ± 17 versus 67 ± 17, P < 0.0001, T-test, df = 195) and there was no or minimal correlation of TSCS score with age or SES. Based on an *a priori *cut-off, the sensitivity and specificity of the TSCS score in this sample were 0.83 and 0.87, respectively. The other half of the population formed a prospective replication sample in which the sensitivity and specificity of the TSCS score were each 0.78. The formula for the TSCS score was similar to the previously published discriminant function analysis [23] but was performed on larger populations of TS and control subjects.

**Table 1:** Components of the TSCS. Weighting coefficients of each variable in the discriminant function are indicated. SS denotes standardized score.

**Cognitive Domain**	**Variable**	**Ref.**	**Weighting Coefficient**
Visual-Motor Ability	Rey-Osterrieth Complex Figure-copy	[51, 52]	1.06057
	Developmental Test of Visual-Motor Integration [SS]	[53]	0.31963
	WAIS-III: Object Assembly [SS]	[54]	-1.47231
	WAIS-III: Block Design [SS]	[54]	0.98230
	WAIS-III: Coding/Digit Symbol [SS]	[54]	-1.47952
	The Pursuit Rotor Dominant Time Off Target	[55]	0.07761
	The Pursuit Rotor Distance	[55]	7.62332
	Judgment Of Line Orientation [# Correct]	[56]	-0.66818
	Money Street Map [Errors Towards]	[57]	3.35712
Spatial-Perceptual Ability	WAIS-III Picture Completion [SS]	[54]	-0.22349
	Kaufman Gestalt Closure [% Correct]	[58]	0.17538
	The Visual Object and Space Perception Battery-Memory Span [SS]	[59]	0.72039
	Test of Facial Recognition [# correct]	[60]	4.22581
Spatial-Relational Memory	The Wechsler Memory Scale-R: Visual Memory [SS]	[54]	2.65698
	Rey-Osterrieth Complex Figure – Immediate Recall	[51, 52]	-0.09704
	Rey-Osterrieth Complex Figure – Delayed Recall	[51, 52]	-0.08993
	Warrington Memory Test (Faces) [# correct]	[59]	1.66476
Working Memory	WAIS-III: Digit Span-Backwards [SS]	[54]	-1.22922
	WAIS-III: Arithmetic [SS]	[54]	-1.09126
	WIDE Range Achievement Test-3-Arithmetic [SS]	[61]	0.72213
Attention-Impulse Control	Test of Variables of Attention 2^nd ^[Commission errors]	[62]	-2.12936
	Matching Familiar Figures [# correct]	[63]	0.02191
Executive Function	Verbal Fluencies: Phonemic [# correct]	[64, 65]	0.45404
	Verbal Fluencies: Semantic [# correct]	[64, 65]	0.24121
	Rey-Osterrieth Complex Figure – Organization	[51, 52]	-0.71923
	The Tower of Hanoi	[66]	0.02541

Stability of the TSCS score was demonstrated by analysis of the TSCS score results for a different group of TS subjects (n= 29), evaluated at baseline and again, one year later. The mean TSCS scores were highly consistent, 48.9 ± 16.9 (baseline) and 49.9 ± 16.1 (one year later). The Pearson correlation of results from the two sets of scores was r = 0.84, df = 27, P < 0.0001). Thus, the TSCS score has test-retest reliability and long-term stability with repeat testing.

### Statistical analyses

Results are presented as mean ± standard deviation (SD). Comparisons among means for more than two groups were performed using an analysis of variance (ANOVA), and Tukey's Standardized Range test (Tukey test) was used for pairwise, post-hoc comparisons. T-tests were also used when comparisons were performed between two groups which were not subgroups in the overall ANOVA comparisons. Correlations between age, height and ovarian status with TSCS score were examined using the Pearson correlation coefficient. Results were considered statistically significant at P < 0.05. All analyses were performed using SAS version 8.2 (Cary, NC).

## Results

### Study population

The genetic study population included 47 women, ages 17–55 years, with partial monosomy for Xp or Xq due to terminal or interstitial deletions, unbalanced translocations, or other rearrangements (Table 2). Comparison groups included adult women with 45, X TS and normal female adult controls. These were mostly the same subjects used in the construction and testing of the TSCS score, which was described above.

**Table 2:** Cytogenetic and phenotypic data for subjects with X chromosome deletions.

**Subject**	**age (yr)**	**ovarian failure**	**karyotype**	**height SD score**	**X inactivation ratio**
			**Xp deletions**		

447	30	no	46, XX	-2.01	57:43
174	22	no	46, X, der(X)t(X;acrocentric) (p22.3;p11.2)mat	-2.59	71:29
175	40	no	46, X, der(X)t(X;acrocentric) (p22.3;p11.2)	-2.2	66:34
482	28	no	46, X, del(X)(p22.33)	-1.10	61:39
428	20	no	46, X, del(X)(p22.33) in affected relative	-0.82	51:49
430	47	no	46, X, del(X)(p22.33)	0.44	100:0
879	38	no	46, XX in affected relative	-2.4	not informative
746	47	no	46, X, del(X)(p22.3)	-2.39	92:8
298	44	no	46, X, del(X)(p22.33p22.33) in affected relative	-0.70	65:35
157	42	no	46, X, del(X)(p22.31p22.33)	-0.75	100:0
217	29	no	46, X, der(X)t(X;Y)(p22.3?1;q11.2) .ish der(X)KAL+STS+DYZ1+DYZ3-DYZ2-	-2.77	93:7
702	39	no	46, XX	-1.68	75:25
466	49	no	46, X, add(X)(p22.31)	-0.61	60:40
439	31	yes	46, X, der(X)t(X;Y)(p22.3;q11.2)	-1.68	89:11
451	40	no	46, X, der(X)t(X;Y)(p22.3;q11.2)	-3.24	68:32
884	43	no	46, X, der(X)t(X;X)(p22.1;q24)	-2.25	100:0
144	36	no	46, X, del(X)(p22.1)	-3.2	100:0
46	34	no	46, X, del(X)(p21.2)	-0.8	100:0
122	17	yes	46, X, del(X)(p21.2)	-2.5	ND
71	20	yes	46, X, del(X)(p11.2)	-1.1	100:0
211	20	yes	46, X, del(X)(p11.23)	-2.4	100:0
324	54	yes	46, X, del(X)(p11.2)	-3.42	100:0
539	23	yes	46, X, del(X)(p11.22)	-2.2	100:0
111	20	yes	46, X, del(X)(p11.21)	-1.1	ND
85	45	yes	46, X, del(X)(p11.2)	-2.8	100:0
109	46	yes	46, X, del(X)(p11.1)	-3.9	100:0
315	23	yes	46, X, der(X)t(X;1)(p11;q44)mat	-3.19	100:0
105	31	yes	46, X, der(X)t(X;1)(p11;q44)mat	-3.18	100:0

			**Xq deletions**		

495	19	yes	46, X, del(X)(q21.1)	-0.86	100:0
383	24	yes	46, X, der(X), t(X, X)(q13.1;p11.21)	3.24	not informative
340	26	yes	46, X, rec(X)dup(Xp), inv(X)(p21q21)	3.17	100:0
403	32	yes	46, X, del(X)(q21.2)	-2.22	100:0
785	37	yes	46, X, del(X)(q22.2)	0.35	100:0
140	37	yes	46, X, del(X)(q21.2)	2.9	100:0
173	48	yes	46, X, del(X)(q13q27.2)	0.73	100:0
475	20	yes	46, X, del(X)(q21)	-2.18	not informative
162	40	yes	46, X del(X)(q21.2)	-2.45	100:0
540	18	yes	46, X, der(X)t(X;Y)(q22;q11.2) .ish der(X)t(X;Y)(wcpX+, wcpY+)	-1.98	100:0
218	35	yes	46, X, del(X)(q22)	-1.38	100:0
139	24	yes	46, X, del(X)(q22)	-1.7	100:0
184	31	no	46, X, del(X)(q24)	-1.53	100:0
314	32	yes	46, X, del(X)(q25)	0.88	100:0
172	34	yes	46, X, der(X)t(X;13)(q22.3;q14.1) .ish der(X)t(X;13)(wcpX+;wcp13+)	-0.73	100:0
192	29	yes	46, X, del(X)(q21.2, q26)	-1.72	100:0
207	44	yes	46, X, del(X)(q22.3;q24)	1.37	100:0
438	40	no	46, X, del(X)(q24q26.1)	0.53	100:0
164	38	no	46, X, del(X)(q24q26)	0.42	100:0

The groups (partial monosomy for Xp or Xq, 45, X TS, and normal controls) were well matched for age (Table 3). The study population (partial X deletions) included Caucasian (39), African-American (2), Hispanic (4), and Other (2). Six subjects were members of kinships (430, 428 and 175, 174 were mother and daughter pairs; 105, 315 were a sibling pair. All other subjects were unrelated.

**Table 3:** Mean age, height standard deviation score, and TSCS score of subjects grouped according to X chromosome abnormality. Data shown are mean ± standard deviation.

	**n**	**Age, yr**	**Height SD score**	**TSCS score**
45, X	127	33.0 ± 10.4	-2.7 ± 1.1	58.3 ± 17.5
controls	104	29.8 ± 9.7	0.1 ± 1.0	68.7 ± 17.5
Xp deletion	28	34.1 ± 10.7	-2.1 ± 1.1	53.1 ± 20.8
<Xp22.3	15	36.3 ± 9.1	-1.6 ± 1.0	54.6 ± 22.0
>Xp22.3	13	31.7 ± 12.2	-2.5 ± 1.0	51.3 ± 20.2
Xq deletion	19	32.4 ± 8.3	-0.2 ± 1.9	68.7 ± 20.4
*SHOX *point mutations	7	42.7 ± 20.2	-2.7 ± 1.6	67.0 ± 11.5

**Figure 1:**
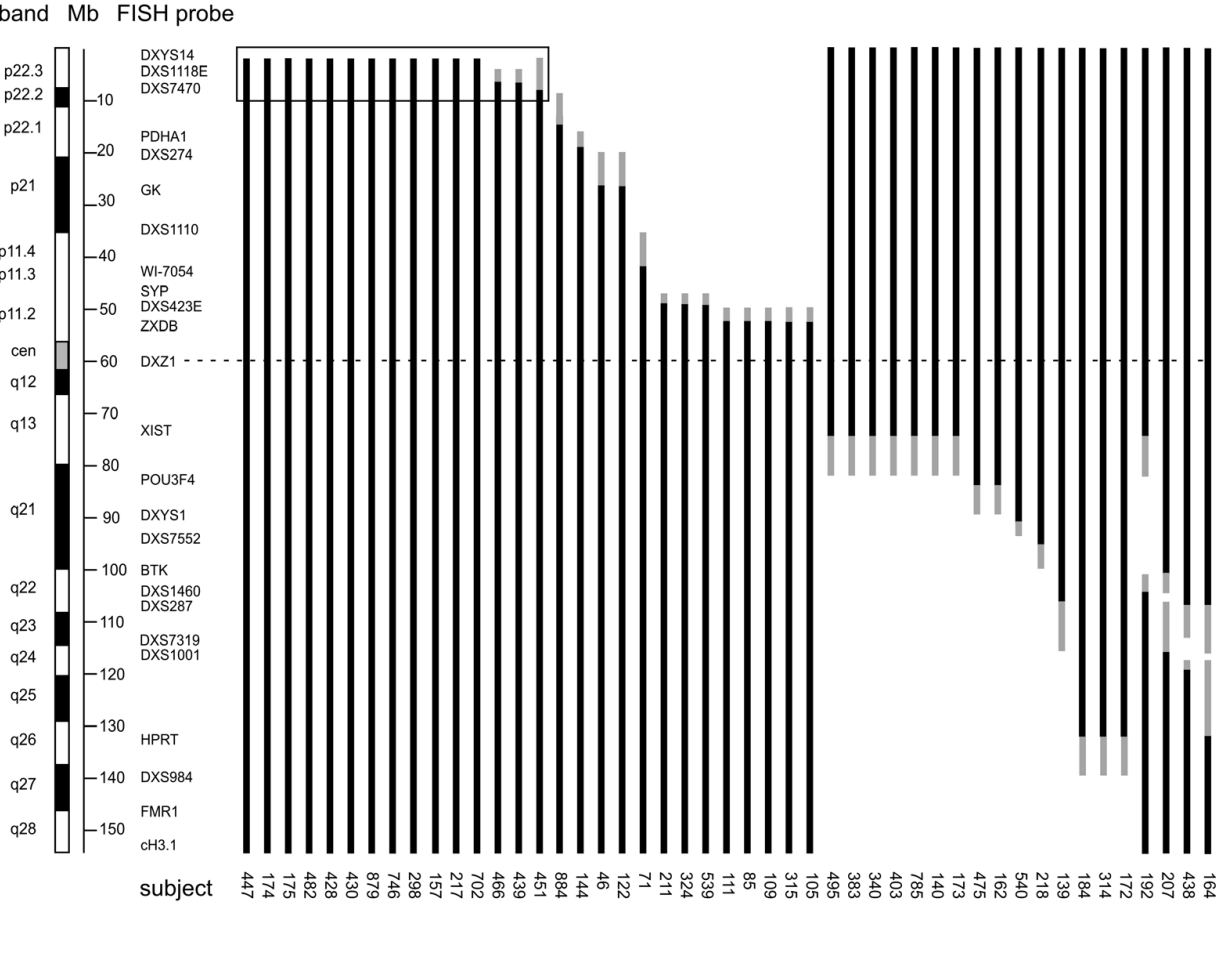
**Schematic depiction of partial X deletions**. Black bars indicate nondeleted regions of the X chromosome; gray bars denote regions of uncertainty between FISH probes. Deletions are indicated by absence of bars. Locations of FISH probes on cytogenetic and physical maps is shown on the left. Xp22.3 deletion breakpoints shown in greater detail in Fig. 2 are boxed.

**Figure 2:**
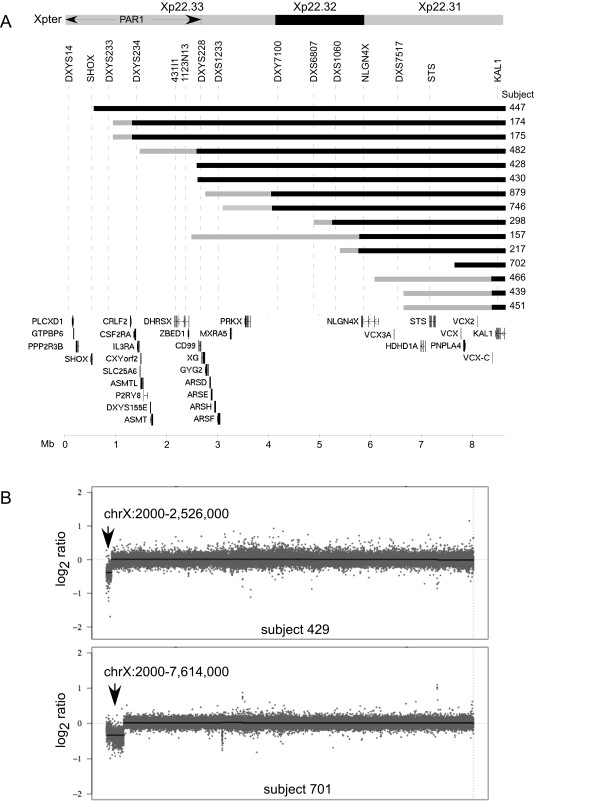
**Refined mapping of Xp22.3 deletions**. a. Positions of microsatellite and FISH markers used to map breakpoints are indicated below ideogram. Black bars denote nondeleted X chromosome segments; gray bars denote segments whose status is uncertain. Positions of RefSeq  annotated genes [32]) are indicated above physical map scale. Marker and gene locations are from the UC Santa Cruz Genome Browser , March 2006 (hg18) assembly. b. High resolution mapping of deletions in subject 429 (son of 430) and subject 701 (son of 702) by array comparative genomic hybridization using an X chromosome tiling array. Each dot represents mean log_2 _signal intensity ratio of probes within consecutive 4000 bp intervals.

X chromosome deletions of the 47 subjects with partial monosomy X are indicated schematically in Figs. 1 and 2. Fifteen of these subjects were previously reported [26,29]. Based on combined karyotype and molecular analyses, 30 subjects had simple terminal deletions, 4 had interstitial Xq deletions, and 13 had complex rearrangements, mostly unbalanced translocations.

### Phenotypes

Table 2 shows phenotypic data for the 47 individual partial monosomy X subjects, including height z-score, ovarian status, and X inactivation status. We calculated the mean TSCS scores for four subgroups: subjects with Xp deletions; subjects with Xq deletions; subjects with 45, X TS; and normal controls. We also examined seven adult subjects who carried *SHOX *point mutations (Table 3).

**Figure 3:**
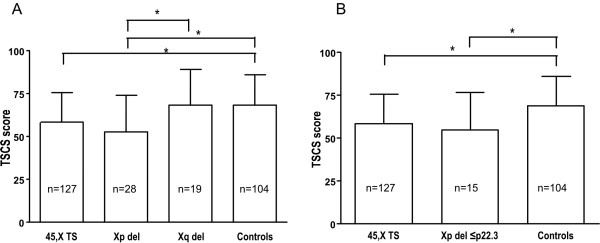
**TSCS scores of subjects according to deletion**. a. Comparison of mean TSCS scores of subjects with any Xp deletion, any Xq deletion, TS, and controls. b. Comparison of mean TSCS scores of subjects with Xp22.3 deletions, TS, and controls. Data shown are mean+SD. *P < 0.05, Tukey's test.

The mean TSCS scores (Table 3; Fig. 3A) differed significantly among the six groups: five with X chromosome or *SHOX *abnormalities and the controls (P < 0.0001, ANOVA, df = 279). The mean TSCS score of the Xp deletion group was similar to that of the 45, X TS group and differed from that of the Xq and control groups (P < 0.05, Tukey test). Thus, deletion of Xp may be necessary and sufficient for the defined TS neurocognitive phenotype. By contrast, it appears that Xq deletions did not affect cognitive outcome, since mean TSCS score in this group was similar to that of controls. Xq deletions did not all overlap (Fig. 1). However, the mean TSCS score of just subjects with overlapping terminal Xq deletions (69.5 ± 21.3, n = 15) was also similar to controls (P > 0.05, T-test, df = 117).

Eight of the Xp deletion subjects had unbalanced translocations, and segmental trisomy could affect their phenotype. However, their mean TSCS score was 56.1 ± 18.2, very similar to that of all other Xp deletion subjects (P = 0.64, T-test, df = 26).

Although all subjects with ovarian failure were receiving estrogen replacement at the time they were studied, it is possible that early ovarian failure could have long-term effects on cognitive outcome. Both Xp and Xq deletion groups were heterogeneous for ovarian failure, so we tested for an association between this phenotype and TSCS score among all 47 subjects. The mean TSCS score of subjects with or without ovarian failure did not differ significantly, and in fact trended toward a higher score in subjects with ovarian failure (64.3 ± 4.4, n = 27 versus 52.8 ± 4.3, n = 20; P = 0.07, T-test, df = 45). Seventeen of the subjects without ovarian failure had Xp deletions; their mean TSCS score was 51.9 ± 19.7, which was similar to the other Xp deletion subjects (P = 0.7, T-test, df = 26).

TS neurocognitive deficits are consistent across a wide age range. There was no significant correlation between age and TSCS score (Pearson r = 0.006, P > 0.9, df = 45) among our 47 subjects with partial X deletions.

Having found the TSCS score associated with deletion of Xp and not Xq, we attempted to narrow the associated region. We calculated the mean TSCS score for the 15 subjects whose deletions were limited to Xp22.3 (up to but not including the *KAL1 *gene at 8.3 Mb) versus the populations of 45, X TS and controls (Fig. 3B). The mean TSCS score was significantly lower in the population deleted for only Xp22.3 compared to the normal control population (P < 0.05, Tukey test, df = 243) and was similar to that of the TS population (P > 0.05, Tukey test, df = 243)

The known TS short stature gene *SHOX *is located in Xp22.3. To determine if *SHOX *might also influence cognition, we evaluated the relationship between stature, deletion of *SHOX*, and TSCS score. There was a correlation between TSCS score and height among the whole population of Xp and Xq deletion subjects (Pearson r = 0.3, P < 0.04, df = 45). However, there was no significant relationship between TSCS score and height among subjects missing *SHOX *(Pearson r = 0.12, P > 0.56, df = 5). As noted above, subjects with Xp deletions >Xp22.3, with mean height SDS -2.5, showed a mean TSCS score similar to that of subjects with deletions limited to Xp22.3, with mean SDS -1.6 (Table 3). The correlation between height and TSCS score observed among all Xp and Xq deletion subjects is likely due to Xp22.3 deletions that encompass both *SHOX *and the cognitive locus rather than a causal association of stature *per se *on cognition.

In addition, we determined the TSCS scores for seven subjects with loss-of-function *SHOX *point mutations, six with *SHOX *haploinsufficiency (dyschondrosteosis) and one with complete absence of *SHOX *(an 84-year old woman with Langer mesomelic dysplasia due to compound heterozygosity) [31]. If *SHOX *haploinsufficiency was responsible for the neurocognitive phenotype, their mean TSCS score should be lower than that of controls and similar to that of the 45, X TS population. The mean TSCS score of the seven *SHOX *mutation carriers was similar to that of controls but was not significantly different from that of the 45, X TS population (Table 3, P >0.05, Tukey test, df = 279) due to the small sample size of the *SHOX *group.

### Refined mapping of Xp22.3 deletions

We fine-mapped deletion breakpoints in the 15 subjects with deletions limited to Xp22.3 (Fig. 1, boxed) using additional FISH probes and/or polymorphic microsatellite markers. The smallest deletion (in subject 447) was mapped using a somatic cell hybrid as described elsewhere [29]. Fig. 2A shows the markers used for mapping and the extent of distal Xp deletions in relationship to the cytogenetic and physical maps of this region of the X chromosome and genes annotated in the RefSeq database [32].

We used array comparative genomic hybridization (CGH) using an X-chromosome specific tiling oligonucleotide array (Nimblegen Systems, Inc.) to confirm and refine the mapping of two deletions associated with mental retardation in male relatives (Fig. 2B). The deletion in subjects 428 and 430, mapped in male relative 429, was visible cytogenetically (Table 2), despite being limited to pseudoautosomal sequences. The deletion was larger in subject 702, spanning the *STS *gene, and was associated with X-linked ichthyosis in her son (701); the mother's deletion was missed by conventional karyotyping (Table 2). In both cases, CGH data were consistent with the results from FISH and genotyping analyses.

Deletion breakpoints were distributed throughout Xp22.3, without any obvious recombination hotspots (Fig. 2A). Six breakpoints fell within the Xp-Yp pseudoautosomal region (PAR1). The smallest deletion, in subject 447, encompassed coding sequences of only four genes: *PLCXD1*, *GTPBP6*, *PPP2R3B*, and *SHOX*. This subject, who was ascertained on the basis of dyschondrosteosis, had a TSCS score of 43.6. Other subjects with PAR1 deletion breakpoints had TSCS scores of 56.1, 21.5, 71.9, 63.9, and 28.1. While the mean for these six subjects differed from that of normal controls (47.5 ± 20, n = 6 vs. 68.7 ± 17.5, n = 104, P = 0.02, T-test, df = 108), we interpret this result from a small cohort cautiously.

### X inactivation

We tested the pattern of X inactivation in blood DNA from our subjects to see if nonrandom X inactivation might play a role in the neurocognitive phenotype. Forty-four of 47 subjects tested were informative for the androgen receptor polymorphism used for the inactivation assay. All informative subjects with deletions extending beyond Xp22.3 had completely skewed X-inactivation, with the deleted X presumably inactive (Table 2). By contrast, 11 of the 15 subjects whose deletions were limited to Xp22.3 (Fig. 1, box) had inactivation patterns ranging from 50:50–90:10, and only four showed >90% skewing (Table 2). As previously noted, the distributions of TSCS scores did not differ between subjects with deletions limited to Xp22.3 versus subjects with larger Xp deletions (Fig. 2B), although the two groups had very different proportions of skewed inactivation. Thus X inactivation skewing did not seem to affect the TSCS score.

## Discussion

An earlier descriptive study suggested that Xp22.3 contains one or more genes that influence cognitive ability in Turner syndrome [22]. The current results provide additional support for this hypothesis. The strength of the conclusion is based on a larger, all adult study population that included both Xp and Xq deletion subjects, with uniform estrogen replacement status for those with ovarian failure. Additionally, the previous study defined a critical region by determining the minimal overlapping deletion among a few individuals with the TS neurocognitive phenotype, assigned as a dichotomous trait. The present study used a quantitative measure of the TS neurocognitive phenotype, the TSCS, to test for associations with deletions, reducing error due to variability of individual results.

The TSCS score is a composite, quantitative summary that partially describes the TS cognitive phenotype. For each individual, the score represents a weighted pattern of performance on a number of cognitive tests that assess multiple clinical domains. Therefore, the mean TSCS score for a group is an average of performance on this composite measure. As with all computed means, the mean TSCS score is not meant to reflect a typical performance but rather the mean of performances by individuals in that population that may contain both typical subjects and atypical subjects. There is overlap in TSCS scores among the TS and control populations. However, the difference in the means is statistically significant (P < 0.001). This overlap in TSCS scores indicates that many TS subjects function within the normal range (mean ± 2 SD for controls). If there were distinct and complete separation of both populations in any cognitive trait, then discriminant function analysis would not be necessary. This cognitive result is similar to the findings for the height deficit in TS. Although there are some TS subjects whose height is in the normal range, the mean height is significantly reduced in TS [1].

The mean TSCS score from 28 women with overlapping Xp deletions of varying size was similar to that of 45, X TS women and differed significantly from that of controls and women with Xq deletions (Fig. 3A). Their overlapping Xp deletions all included Xp22.3, the region previously hypothesized to affect TS cognition. We therefore investigated the mean TSCS score of the 15 subjects whose deletions were restricted to this interval (<Xp22.3). Their mean TSCS score was also similar to that of 45, X TS subjects and differed significantly from that of controls (Fig. 3B). Larger Xp deletions (>Xp22.3) were not associated with further reduction in mean TSCS score or increased individual variability in performance (Fig. 3B, Table 3). This supports the conclusion that deletion of Xp22.3 may be sufficient for producing this aspect of the neurocognitive profile in TS.

Previous studies have shown that the likelihood of ovarian failure in patients with terminal Xp deletions is directly related to the size of the deletion [26,33]. Our data also show this relationship. The mean TSCS score of Xp deletion subjects without ovarian failure was similar to that of 45, X TS subjects, suggesting that distinct Xp loci contribute to ovarian failure and neurocognitive deficits in TS.

Using an expanded panel of FISH and microsatellite markers, and in two cases CGH, we mapped the associated interval to an 8.3 Mb region of Xp22.3 containing approximately 30 genes. It is interesting that a deletion limited to the pseudoautosomal region in subject 430 was associated with mild developmental delay in her son. This is surprising, since haploinsufficiency of the entire pseudoautosomal region does not usually cause global developmental delay in females. Possible explanations include unmasking of a recessive Y pseudoautosomal allele, gender-specific effects, or coincidence. The boy did not undergo detailed cognitive testing.

While it appears that deletions limited to pseudoautosomal sequences may be sufficient to reduce the TSCS score, as previously hypothesized, this conclusion is tempered by the fact that two of these deletions were due to unbalanced Xp;Yq translocations, and Yq heterochromatin could exert position effects on nearby genes such as *NLGN4X *[MIM 300427]. The lack of effect of Xq deletions on the mean TSCS score suggests that the phenotype is specific for deletion of Xp22.3 rather than a nonspecific manifestation of aneuploidy or ovarian failure, which showed no correlation with TSCS score.

Several aspects of the Xp22.3 region are noteworthy. Deletions limited to Xp22.3 usually manifest short stature rather than the full TS phenotype and are associated with variable patterns of X inactivation, whereas larger deletions are associated with nonrandom inactivation of the deleted X chromosome [34]. Patients with Goltz, Aicardi, or MLS syndrome, who may show deletions limited to Xp22.3 with highly skewed inactivation [34], were excluded from our study population. Most of our subjects with deletions limited to Xp22.3 did not show highly skewed inactivation. Subjects with larger Xp deletions or Xq deletions generally showed nonrandom inactivation, presumably of the deleted X chromosome. The TSCS score distributions were similar for subjects with deletions limited to Xp22.3 and non-skewed inactivation and subjects with larger Xp deletions and skewed inactivation. Furthermore, the TSCS scores of subjects with Xq deletions, all of whom showed highly skewed inactivation, differed from those of subjects with Xp deletions. Thus, TSCS score was not related to pattern of X inactivation.

Most of the 30 or so genes in the Xp22.3 critical region (Table 4) escape X inactivation, and many have Y-linked homologs, suggesting that they are dosage sensitive [17]. Functional studies of genes in this region using animal models are hindered by the apparent absence of almost the entire region in mouse and rat, except for two genes, *PRKX *[MIM 300083] and *STS *[MIM 308100] [19].

**Table 4:** RefSeq genes in critical region.

**Gene name**	**RefSeq accession**	**Product/function**
*PLCXD1*	NM_018390	phospholipase
*GTPBP6*	NM_012227	GTP-binding protein-like
*PPP2R3B*	NM_199326	phosphatase regulatory subunit
*SHOX*	NM_000451	transcription factor/chondrocyte growth
*CRLF2*	NM_001012288	cytokine receptor-like
*CSF2RA*	NM_006140	cytokine subunit
*IL3RA*	NM_002183	interleukin 3 receptor subunit
*SLC25A6*	NM_001636	mitochondrial adenine nucleotide translocator
*CXYorf2*	NM_025091	hypothetical protein
*ASMTL*	NM_004192	acetylserine O-methyltransferase-like
*P2RY8*	NM_178129	G-protein coupled purinergic receptor
*DXYS155E*	NM_005088	novel protein
*ASMT*	NM_004043	acetylserine O-methyltransferase
*DHRSX*	NM_145177	dehydrogenase/reductase
*ZBED1*	NM_004729	Ac-like transposable element
*CD99*	NM_002414	cell surface antigen
*XG*	NM_175569	cell surface antigen
*GYG2*	NM_003918	glycogenin
*ARSD*	NM_001669	arylsulfatase
*ARSE*	NM_000047	arylsulfatase (chondrodysplasia punctata)
*ARSH*	NM_001011719	arylsulfatase
*ARSF*	NM_004042	arylsulfatase
*MXRA5*	NM_015419	adlican
*PRKX*	NM_005044	protein kinase; kidney development
*NLGN4X*	NM_020742	neuroligin 4; see text
*VCX3A*	NM_016379	germ cell protein; see text
*HDHD1A*	NM_012080	haloacid dehalogenase-like hydrolase domain
*STS*	NM_000351	steroid sulfatase; see text
*VCX*	NM_013452	germ cell protein; see text
*PNPLA4*	NM_004650	phospholipase
*VCX2*	NM_016378	germ cell protein; see text

*STS *encodes steroid sulfatase, deficiency of which causes X-linked ichthyosis [35]. Mouse *Sts *has been implicated in modulating neurosteroid levels and thus GABA_A _receptor function [36]. Mouse *Sts *is pseudoautosomal and therefore dosage sensitive, and 39, X mice lacking one copy of *Sts *show altered GABA_A _receptor expression [37]. These mice also show reduced visuospatial attention [38]. Restoration of *Sts *diploid dosage by a truncated sex chromosome carrying the pseudoautosomal region and a small number of X-linked genes normalized GABA_A _receptor expression and rescued the visuospatial attention deficit [37,38]. The investigators concluded that haploinsufficiency of a pseudoautosomal gene, possibly *Sts*, is responsible for visuospatial attention deficits in 39, X mice and perhaps analogous neurocognitive deficits in humans with TS.

Other genes in the Xp22.3 region that have been linked to cognitive phenotypes include *VCX3A *and *NLGN4X*. *VCX3A *[variable charged X-linked gene 3A, MIM 300533] belongs to a gene family with multiple closely related members on both the X and Y chromosome. *VCX3A *was proposed to be involved in cognition on the basis of overlapping Xp22.3 deletions associated with mental retardation in a few males [39]. However, a subsequent study found that only one out of seven males in a family with icthyosis due to a microdeletion that included *VCX3A *and *VCX *[MIM 300229] had mental retardation [40]. Furthermore, expression of human *VCX *genes is restricted to male germ cells [41].

*NLGN4X*, also in Xp22.3, encodes a neuroligin, or neural cell adhesion molecule, widely expressed in brain that escapes inactivation and has a functional Y homolog [17,42]. Overexpression of the protein in cultured hippocampal neurons has been shown to stimulate formation of presynaptic terminals [43]. Frameshift and nonsense mutations in *NLGN4X *have been linked to Asperger syndrome/autism [MIM 300495, 300497] [44] and X-linked mental retardation [MIM300495] [45]. Although cognitive effects of reduced *NLGN4X *dosage have not been described in female carriers of these mutations, autistic features have been described as part of the 45, X TS neurocognitive phenotype [46], and three females with autism and deletions of distal Xp encompassing *NLGN4X *have been reported [47]. However, none of our subjects carried a diagnosis of autism spectrum disorder, and the relationship of TSCS results and autistic features is not clear. The TSCS score is based on performance on cognitive tests and therefore has no direct relationship to autistic behavior. However cognitive impairment on visual-perceptual and spatial processing tasks may be related to increased risk of social problems and altered interpersonal relationships.

Our study has several limitations. First, nonmosaic partial X chromosome deletions are rare. In order to obtain sufficient sample size for statistical comparisons, we included eleven subjects with unbalanced translocations (eight with Xp deletions and three with Xq deletions). While none of these subjects had any obvious features of autosomal trisomy, presumably due to spread of X inactivation to the autosomal segment, we cannot exclude the possibility of cognitive effects. However, the mean TSCS score of the unbalanced translocation subjects with Xp deletions was very similar to that of all Xp deletion subjects. Similarly, because the number of Xq deletion subjects was small, we included subjects whose deletions did not all overlap. This deletion heterogeneity could mask an effect of a locus in Xq. However, the mean TSCS score of just the subjects with terminal Xq deletions was similar to controls. Our results strongly support a TS neurocognitive locus in Xp but do not absolutely exclude a neurocognitive effect of deletion of Xq.

There was ascertainment bias toward ovarian failure, particularly for subjects with Xq deletions, although this aspect of the TS phenotype did not show any correlation with TSCS score. Although the study included only adults, the age range was relatively broad (17–55 years). However, age did not correlate with TSCS score either. Because parental DNAs were not available for most of the subjects, we could not address any possible imprinting effect, as has been claimed for social-behavioral aspects of the TS cognitive phenotype [48].

As with any complex trait, the presence and severity of the TS neurocognitive phenotype is variable. This study used discriminant function analysis, a relatively uncommon method of defining cognitive phenotype. This method was used because it allows a quantitative approach to cognition using a group of subjects who, though similar, have a moderate degree of cognitive heterogeneity. There is clearly overlap between the distributions of TSCS scores of 45, X TS versus controls: some controls have lower TSCS scores than the 45, X average, and some 45, X TS subjects have higher TSCS scores than the control average. Because of this overlap, the ability to infer genotype/phenotype correlations based on individual subjects is limited. For this reason, we based our conclusions on mean TSCS scores of groups of subjects with similar deletions. This approach does not require that every individual deleted for the cognitive locus manifest the identical cognitive phenotype.

The TSCS reflects performance in multiple cognitive domains, and includes visual-motor tasks, visual-perceptual tasks, executive function/attention tasks and memory tasks. Given the complex TS cognitive phenotype in TS, it is not expected that the absence of a single gene leads directly to this complete phenotype or even to a specific cognitive trait associated with TS. Therefore, it is unlikely that within Xp22.3 there is a specific gene that codes for a specific cognitive dysfunction. Rather, absence of this gene most likely affects a biological activity that is intimately involved in the development of the phenotype, manifesting as a complex and nonfocal neurocognitive phenotype. The TSCS is still an incomplete representation of the TS cognitive phenotype. There are other cognitive features, including motor and social function not included in the TSCS. Therefore, it is likely that the TS cognitive phenotype is due to multiple cognitive determinants and multiple genes, each contributing to the phenotypic variance.

Molko et al. [49] described cerebral structural changes in TS compared to female controls. These regions included the right intrapatietal sulcus and bilateral superior temporal sulci. These regions have classically been associated with spatial-perceptual ability and spatial-relational memory. Dysfunction in these cognitive domains is part of the TS cognitive phenotype. Therefore, it is reasonable to speculate that the cognitive change associated with deletion of Xp22.3 may be mediated through structural changes in these anatomic regions.

These results are clinically relevant for patients with partial X chromosome deletions. TS is classically defined as monosomy X with typical physical and cognitive phenotypes. Early karyotype/phenotype correlations studies implicated loss of the short arm in growth failure. The discovery of the *SHOX *gene suggested a continuum between idiopathic short stature [MIM 300582], due to deletion of only the *SHOX *gene, and TS, which could be viewed as a contiguous gene deletion syndrome. For purposes of diagnosis and treatment, the distinction has been made between deletions smaller than Xp22.3 associated only with short stature (idiopathic short stature or dyschondrosteosis [MIM 127300]) and larger Xp deletions associated with both short stature and ovarian failure (TS) [50]. However, it must now be recognized that patients with Xp22.3 deletions, regardless of diagnosis, are at risk for TS neurocognitive deficits and should be evaluated accordingly. Furthermore, in the absence of mosaicism, the risk of these cognitive deficits in patients with Xq deletions is low.

## Conclusion

In summary, haploinsufficiency of genes located in Xp22.3 appears to cause at least part of the multifaceted cognitive phenotype of 45, X TS, as indexed by TSCS score. This critical region contains 30 or so genes. The lack of murine orthologs of most of these genes (with the exception of *STS *and *PRKX*) and the limitations of mouse models for complex human cognitive abilities precludes identifying the causative gene by knockout models. An alternative approach is to test for association between genetic variation in Xp22.3 genes and the TSCS in 45, X TS subjects. In addition, high resolution genomic technologies such as array comparative genomic hybridization may detect submicroscopic deletions associated with TS cognitive deficits that would identify the causative gene(s), as was the case for short stature due to *SHOX *deletion. Identification of the TS cognitive gene would facilitate early diagnosis and intervention in individuals with the associated neurocognitive deficits.

## Competing interests

The author(s) declare that they have no competing interests.

## Authors' contributions

ARZ and JLR conceived of the study, participated in its design and coordination and wrote the manuscript. DR participated in the interpretation of the results and manuscript preparation. GS participated in the design of the neurocognitive assessment and manuscript preparation. PR performed molecular genetic studies. FE performed karyotypes. HK performed statistical analyses. KK coordinated subject recruitment and supervised neurocognitive testing. All authors read and approved the final manuscript.
